# The Multimedia activity recall for children and adolescents (MARCA): development and evaluation

**DOI:** 10.1186/1479-5868-3-10

**Published:** 2006-05-26

**Authors:** Kate Ridley, Tim S Olds, Alison Hill

**Affiliations:** 1 School of Education, Flinders University, Adelaide, Australia; 2 School of Health Sciences, University of South Australia, Australia

## Abstract

**Background:**

Self-report recall questionnaires are commonly used to measure physical activity, energy expenditure and time use in children and adolescents. However, self-report questionnaires show low to moderate validity, mainly due to inaccuracies in recalling activity in terms of duration and intensity. Aside from recall errors, inaccuracies in estimating energy expenditure from self-report questionnaires are compounded by a lack of data on the energy cost of everyday activities in children and adolescents. This article describes the development of the Multimedia Activity Recall for Children and Adolescents (MARCA), a computer-delivered use-of-time instrument designed to address both the limitations of self-report recall questionnaires in children, and the lack of energy cost data in children.

**Methods:**

The test-retest reliability of the MARCA was assessed using a sample of 32 children (aged 11.8 ± 0.7 y) who undertook the MARCA twice within 24-h. Criterion validity was assessed by comparing self-reports with accelerometer counts collected on a sample of 66 children (aged 11.6 ± 0.8 y). Content and construct validity were assessed by establishing whether data collected using the MARCA on 1429 children (aged 11.9 ± 0.8 y) exhibited relationships and trends in children's physical activity consistent with established findings from a number of previous research studies.

**Results:**

Test-retest reliability was high with intra-class coefficients ranging from 0.88 to 0.94. The MARCA demonstrated criterion validity comparable to other self-report instruments with Spearman coefficients ranging from rho = 0.36 to 0.45, and provided evidence of good content and construct validity.

**Conclusion:**

The MARCA is a valid and reliable self-report questionnaire, capable of a wide variety of flexible use-of-time analyses related to both physical activity and sedentary behaviour, and offers advantages over existing pen-and-paper questionnaires.

## Background

The extent of equivocal research findings related to the associations between physical activity, sedentary behaviour, and overweight may be associated with inadequate measures of physical activity. It has been demonstrated that the type of physical activity measure used in research studies can be critical when attempting to establish relationships between markers of health and physical activity [1]. In order to address and further understand the health outcomes associated with the prevalence of overweight and sedentary behaviour, it is essential that trends in physical activity and participation in various specific behaviours be accurately measured in a variety of populations. As some cardiovascular disease risk factors and related behaviours have foundations in childhood and adolescence [2,3], this age group is of particular interest.

As physical activity is a multi-dimensional construct that can vary in terms of type, duration, intensity and frequency, it is difficult to measure, especially in children and adolescents. At present there is no "gold standard" measure of physical activity that can accurately measure each of these four dimensions of physical activity. High day-to-day variability in activity level, particularly in children, can further contribute to measurement error when assessing habitual activity [4]. A number of different methods can be used to estimate physical activity levels, ranging from precise measures of energy expenditure (EE), such as doubly labeled water, to objective measures of physical activity, such as accelerometers, to more subjective measures of physical activity, such as self-reports [5]. Each method has its own advantages and limitations in terms of validity, cost, versatility, and practicality in free-living situations. At present researchers are using a wide variety of these instruments, however as the methods measure different constructs of physical activity, comparisons between studies are often difficult, if not impossible.

The use of self-report questionnaires are often favoured in large-scale surveys due to their low cost and convenience. However, there is considerable variation in the design of these instruments. For instance, the definition of activity varies across self-reports. While some questionnaires focus on sports or leisure time physical activity only [6], other questionnaires also measure other forms of activity, such as sedentary behaviours, that may have independent relationships with obesity and health [7,8]. In addition, physical activity correlates (e.g. family and peer relations, associations with the built environment, etc.) may be related to specific activities, or times of the day. These characteristics of behaviour are best measured with use-of-time instruments, where activities are recalled sequentially across an entire day. The lack of understanding about what influences physical activity and sedentary behaviour in children may be related to the widespread ineffectiveness of physical activity interventions in children [9]. Equally, the evaluation of interventions may also be hampered by inadequate measures of physical activity.

Self-report questionnaires use a variety of recall periods, ranging from one day to weekly, monthly or yearly estimates of activity. While self-report questionnaires generally exhibit good test-retest reliability, self-reports have traditionally been limited by low to moderate validity, particularly in child and adolescent populations. Validity studies in children typically report validity coefficients ranging from r = 0.20 to 0.60 [5,10]. Although this may be due to the lack of a "gold standard" method of comparison, low validity is also largely due to limitations in the use of self-report questionnaires with children. Children often struggle when using recall instruments: they tend to elide (i.e. merge together) experiences, have trouble remembering a whole day, have a poor sense of duration, have difficulty determining intensity of activity, and often lack motivation to complete the task [10-13]. Due to these recall problems, self-report questionnaires that ask subjects to recall "usual", or "typical" activity, or periods > 7-d, using activity checklists generally report lower validity coefficients than those with shorter recall periods [5,10].

Aside from recall errors, inaccuracies in estimating energy expended during habitual physical activity are compounded by a lack of energy cost data on children's activities. Many questionnaires use compendia of energy costs to convert self-report data into EE [14,15]. However, energy cost per unit body weight tends to decrease as age increases [16]. While much work has been done on developing compendia of energy costs for adults [17], there has been little systematic research into the energy cost of children performing adult-type activities (e.g. household chores) or child-specific activities, such as playground games.

It is essential that valid, reliable and versatile self-report measures of physical activity, specifically designed for children and adolescents, be developed. Such instruments may be used to track historical trends and to compare subgroups, providing information for designing and evaluating physical activity interventions with greater leverage. In order to improve on existing self-report questionnaires, the structure of recall instruments need to be designed to optimise children's ability to recall activity. This involves the integration of characteristics that are known to enhance recall, such as a 1-d recall and segmented day format [12,18]; and the development and trialing of new strategies. For example, computer-delivered instruments have recently shown potential in enhancing children's recall and increasing motivation [19,20]. However, few computer-delivered instruments have capitalised on the capability of multimedia to incorporate characteristics such as sound, video, automated checks, or to facilitate flexible, varied and easily obtainable analyses.

The Multimedia Activity Recall for Children and Adolescents (MARCA), a use-of-time self-report instrument was designed to address both the limitations of self-report recall questionnaires and the lack of energy cost data in children. The purpose of this article is to describe the development and evaluation of the MARCA. The MARCA was evaluated in terms of: (a) test-retest reliability; (b) criterion validity; and (c) content/construct validity. Criterion validity involves comparing the results from a self-report questionnaire with a comparative measure, in this case, accelerometer counts. Content validity refers to how well the individual variables measured by the questionnaire are a representative sample of the universal behavioural domain being assessed, in this case, activity behaviour [21]. Once the variables to be measured have been established, construct validity refers to the degree of confidence that the information provided by the questionnaire reflects these variables. Therefore, the aim of the content/construct validity analyses was to assess whether data collected using the MARCA exhibited relationships and trends in children's physical activity consistent with established findings from a number of previous research studies.

## Methods

### Participants and sampling

Convenience samples were chosen for the reliability (n = 32) and the criterion validity study (n = 66). Children between the ages of 9.0 and 13.5 years were recruited from local primary schools. The participants in the content/construct validity study (n = 1429) were children aged between 9.0 and 15.0 years recruited from randomly selected schools in South Australia. For each of the studies, schools, parents and children were sent letters inviting them to participate. The protocol for each study was approved by the ethics committees of the University of South Australia and the Department of Education and Children's Services. Informed consent was obtained from the schools and parents of the children involved. Characteristics of the three samples are shown in Table 1.

**Table 1:** Sample characteristics (sample size, gender, age, and BMI) for the MARCA reliability and validity studies.

**Study**	**n**	**Gender**	**Age (y)**	**BMI (kg/m^2^)**
test-retest	32	14 F/18 M	11.8	18.9
reliability study			*0.7*	*3.5*
criterion	66	33 F/33 M	11.6	19.5
validity study			*0.8*	*3.2*
content/construct	1429	729 F/700 M	11.9	19.7
validity study			*0.8*	*3.8*

Note: Upper value = mean; lower value = standard deviation; BMI = body mass index.

### Measures

#### Description of the MARCA

The MARCA is a 1-d self-report recall questionnaire administered by computer, consisting of three modules, a 1-d activity recall, a compendium of child-specific energy costs, and an analytical module. The MARCA aims to be a self-report questionnaire that:

(a) accurately measures use-of-time and provides good estimates of daily EE (as a multiple of resting metabolic rate) in children;

(b) provides sufficient detail on type, duration, intensity and frequency of activity;

(c) yields varied and flexible outcome measures capable of addressing a number of research questions;

(d) addresses all forms of activity, i.e. organised and non-organised physical activity, sedentary activity, incidental activity, etc.;

(e) covers a recall period that best increases the likelihood of valid data;

(f) minimises researcher burden associated with data entry, analysis and storage;

(g) is a simple and interesting task for children to complete.

The MARCA asks children to recall their previous day's activities in time slices of 5 min or more. A 1-d recall period was chosen because children's recall has been shown to be more accurate over this period than longer periods of recall [12,18]. The minimum 5 min resolution was chosen to allow for a number of short duration activities to be recalled, e.g. talking on the phone or walking to a friend's house. Short segments of recall are especially important in questionnaires for children as their activity tends to be characterised by short bursts of incidental activity [22]. As recent research suggests, short bouts of moderate to vigorous physical activity (MVPA) that accumulate over the day have health benefits [23], it is important that these bouts of activity be recorded.

**Figure 1:**
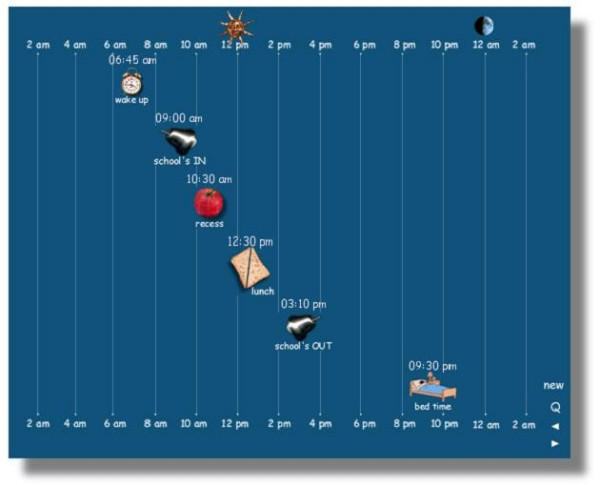
Example of a school day segmented according to school break times.

The MARCA allows children to recall either a school day or another day (weekend, holiday or day off from school). Previous literature suggests that a child's ability to recall their day in terms of type and frequency is enhanced when the day is segmented [12,24]. A segmented day format also allows the activities to be reported in the context within which they have been performed [25]. The MARCA allows segmentation to be individualised for each subject by allowing each child to set activity time anchor-points, i.e. school breaks for school days and meal times for non-school days. The user drags icons along a timeline (Figure 1) to set the anchor points. The sliders do not allow inconsistent settings (e.g. going to bed before dinner). Each of the subsequent MARCA screens then asks the user to report their activity during their individualised segments. These segments are similar to landmarks that resulted in a more comprehensive recall of activity previously identified by McKenna and colleagues in their qualitative interviewing of 8–16 year old children [24].

**Figure 2:**
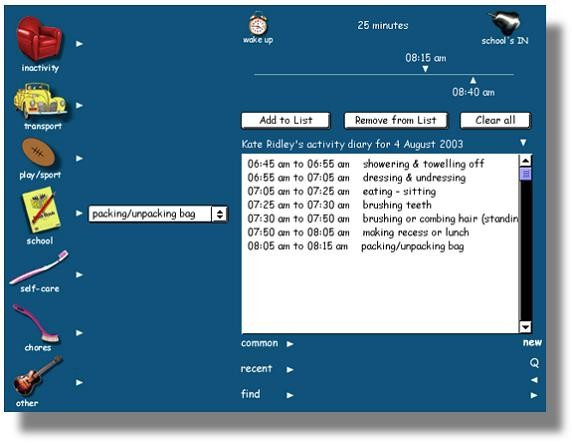
Example of one of the MARCA's segmented day screens.

During each segment children can choose from over 200 activities grouped into seven categories; inactivity; transport; play/sport; school work; self care; chores and 'other'. The 'other' category contains miscellaneous activities, such as playing musical instruments, which do not fit within the described categories. These activities were chosen by scanning the Ainsworth compendium [17] for activities likely to be undertaken by children, reviewing activity lists within existing physical activity questionnaires and reviewing papers that describe common activities performed by children [26,27]. Pilot testing of the MARCA with children has provided the opportunity to evaluate and refine the activity lists by noting activities that children were having trouble locating within the lists, or activities which they indicated were missing, etc. Having chosen an activity, children indicate the duration of the activity by dragging icons along a timeline that increases in 5 min intervals. A slider above the timeline is dragged to the start time of the activity and a slider below the timeline is dragged to the finish time of the activity (see Figure 2). The selection of duration using a scrolling timeline (rather than simply writing a duration in exact minutes into a box) has previously been found to assist children in judging time [20].

Some activities require a subjective intensity ranking. These activities are mostly physical activities such as locomotor activities, games and sports that can be performed at a wide range of intensities. In these cases, an intensity screen asks the user whether the activity was performed at light, medium or hard intensity. As children have difficulty understanding the concept of intensity [28], users are asked whether they need any help to decide. If so, a screen will appear that plays videos of children playing a ball game at varying intensities (see Additional File 1). Trost and colleagues [28] and Tremblay and colleagues [29] concur that videos are effective in helping children understand the concept of physical activity intensity. The videos used in the MARCA were initially developed for use in the Computer-delivered physical activity questionnaire (CDPAQ), a self-report questionnaire developed prior to the MARCA and a more thorough description of their development is available in Ridley et al. [20]. Once an activity has been selected, the name of activity, its identifying code, the start and end time, and duration of activity are recorded. Children continue to add activities to their activity list within each segment of the day, until the end of the day is reached.

The activity recall is linked to a compendium of energy costs. Each activity within the MARCA is assigned an individual code, loosely based on the system used by Ainsworth and colleagues (2000). The MARCA code consists of six digits which provide information about specific characteristics of each activity [e.g. body position when performing the activity (sitting, standing, etc) and intensity of activity]. The code also links the activities to the compendium of energy costs and assists in undertaking a wide variety of flexible analyses. The MARCA compendium was created after a thorough literature search into the energy cost of children's activities [30]. Due to the lack of energy cost data in children, approximately 70% of the activities in the current compendium use MET levels from the adult data presented in the Ainsworth compendium (2000). However, as data become available on child-specific energy costs, they can be updated in the compendium and all previously collected MARCA profiles can be automatically updated.

On completion of the recall, children are able to store their profile (a 'profile' is a recall by one child on one day) to the MARCA program or save the profile to an external text file. Diaries can be reloaded from the program or imported from a text file for modification or analysis. The multimedia environment of the MARCA allows for a number of checks, or alerts, to ensure subjects do not enter invalid data. These checks include: range checks on height, weight, age, etc; overlap checks that ensure two activities are not entered during the same time period; and gap checks ensure there aren't any large gaps of time missing. The MARCA also ensures that a maximum of 1440 min (1-d) of activity are entered. These checks attempt to ensure the data collected are as complete as possible. As the MARCA involves almost entirely point and click entries, lost data due to illegible or incomprehensible responses that can occur with pen-and-paper questionnaires are rare. Researcher burden associated with data entry and analysis is also reduced.

The MARCA's analytical module facilitates analysis of data collected by the 1-d recall. Profiles are imported into the analytical module from MARCA outputs in text files. The analytical module performs a number of data cleaning functions, e.g. locating all instances of 'other' activities and allowing alternatives from the compendium to be chosen (activity and associated energy cost); scanning for evidence of implausible values; etc. These procedures allow researchers to cull profiles where children have not made a genuine effort at recall, and to identify those subgroups in which this is most likely to occur.

Due to the MARCA's activity coding structure, the analytical module can yield a variety of analyses relating to both use of time and EE. The module can calculate: the subject's physical activity level (PAL) – an expression of the multiple of the resting metabolic rate for a day's energy expenditure, calculated as a weighted mean MET.d^-1 ^score; time spent above a given MET level (e.g. time spent in physical activity ≥ 3 METs); and calculate the time spent lying down, sitting, standing or in locomotion. The module can also determine the number of minutes and estimated energy cost for any activity or set of activities for a single profile or any set of profile and calculate the time-distribution of any activity or set of activities (i.e. the percentage of children engaged in any activity or set of activities across the day).

#### Accelerometry

The MTI Actigraph accelerometer (Model AM7164-2.2C, Manufacturing Technologies, Inc., Fort Walton Beach, FL) was used in the criterion validity study. The Actigraph is a small (5.1 × 3.8 × 1.5 cm) one-dimensional accelerometer that measures vertical movement and stores data over user-specified time intervals. The Actigraph has been found to be a valid and reliable tool for assessing children's physical activity [31].

### Procedures

#### Test -retest reliability measurement procedures

Children reported their previous day's activity using the MARCA. Children were not aware they would be asked to re-do the MARCA later in the day. Upon completion, children returned to the classroom where they resumed their usual school activity for 285 min, before being asked to recall their previous day's activities for a second time. Children were given identical instructions during each MARCA administration.

#### Criterion validity measurement procedures

Children were fitted with the Actigraph accelerometer on the day prior to the MARCA recall. The Actigraph was set to 1 min sampling intervals. Children were fitted with the Actigraph at their school at 9 am. The Actigraph was encased in a small, material pouch and attached to the child's right hip on an adjustable cloth belt. Children were given detailed instructions regarding the care of the accelerometer. Children were not informed of the purpose of wearing the accelerometer, or that they would be asked to recall their activity during the next day. Children were instructed to wear the activity monitor until bedtime and only remove when engaging in water activities. Children were provided with a sheet to note when the accelerometer was removed and put back on. On the morning of the next day, the accelerometers were collected and downloaded onto a computer for analysis. Children then completed the MARCA, reporting on their previous day's activities.

#### Construct/content measurement procedures

Each child completed the MARCA on two to five occasions, recalling their previous day's activity. Every child completed the MARCA on at least two days, at least one of which was a full day at school, and at least one a non-school day (weekend, holiday or day off).

In each study the MARCA was completed in the school's computer room under the supervision of trained research assistants. Height and mass were measured using standard protocols and equipment recommended by the International Society for the Advancement of Kinanthropometry [32]. Body Mass Index (BMI: kg/m^2^) was then calculated.

### Data analysis

#### Test-retest reliability data analysis

Test-retest reliability was assessed by intra-class coefficients for PAL, MVPA, defined as ≥ 3 METs, and minutes spent in locomotion. All MARCA variables were skewed and therefore normalised using Box-Cox transformations prior to analysis [33]. Paired t-tests assessed whether there were any significant differences between the trials. Bland-Altman analyses [34] were used to quantify the level of agreement between the repeated measures of the MARCA. A sample size of 32 would be able to detect a reliability correlation of 0.5 with a power of 0.85, and detect a reliability correlation of 0.8 with a power of 1.00.

#### Criterion validity data analysis

Stored minute-by-minute Actigraph accelerometry counts were converted into total activity counts and average counts.min^-1^. The MARCA profiles were uploaded to the MARCA analysis module to calculate PAL, minutes spent in MVPA and minutes spent in locomotion. The MARCA variables were calculated for only the time that the Actigraph was worn. The three MARCA variables were chosen in an attempt to validate the MARCA as a tool capable of estimating EE as a multiple of resting metabolic rate (expressed as PAL), physical activity (MVPA) and use-of-time (minutes spent in locomotion). Time spent in locomotion was chosen due to the properties of the Actigraph accelerometer. As uni-axial accelerometers only measure vertical movement [31], it is likely that some activities that require ≥ 3 METs may not be measured accurately by the Actigraph (notably activities requiring substantial upper body movement and little lower body movement, e.g. some household chores). The Actigraph may therefore be more accurate in measuring time spent in locomotion (cycling excluded). While the majority of locomotion activities are MVPA activities, the MARCA compendium contains 30 activities in the MARCA compendium that are classified as MVPA, but are not classified as locomotion (i.e. they are primarily standing activities). In addition, there are 23 activities not in the locomotion category that are classified as MVPA. Therefore, the MVPA and locomotion variables are sufficiently different to conduct analyses on both.

All Actigraph and MARCA variables were skewed, with the Actigraph total counts unable to be normalised. Therefore Spearman's correlation coefficient was used to assess the relationship between:

a) average Actigraph counts.min^-1 ^and average PAL reported by the MARCA;

b) Actigraph total counts for the entire monitored period (mean = 600 min) and MVPA reported by the MARCA; and

c) Actigraph total counts and time spent in locomotion reported by the MARCA.

Actigraph counts were not converted into MVPA using existing regression equations due to the discrepancy between the various published MVPA cut-points [14,35]. The large standard error of estimates of the existing regression equations [14,35] make it difficult to ascertain whether discrepancy between the MTI and MARCA MVPA minutes is due to misclassification of intensity by the MTI or recall error using the MARCA. With an alpha level of 0.05, a sample size of 66 would be able to detect a validity correlation coefficient of 0.36 with a power of 0.85. In order to investigate the age and gender trends associated with the validity of self-report questionnaires, validity coefficients were also calculated for age (<11 y and ≥ 11 y) and gender subsets.

#### Criterion validity data analysis

The content/construct validity analyses undertaken in this study examine physical activity levels by age and gender. While the analyses provide interesting and legitimate investigations into the activity patterns of South Australian children, the primary aim here is to determine the MARCA's content/construct validity. When more than one school day or non-school day was recalled, values were averaged. The MARCA's analytical module was used to determine an overall daily PAL, minutes in MVPA (activities assigned an energy cost of ≥ 3 METs using the MARCA compendium) and minutes devoted to various activity subsets. The number of minutes spent in the following subsets was calculated:

(a) screen time (time spent watching television or videos, time spent playing video games, and time spent in all forms of computer use, e.g. games, internet, word-processing, etc.);

(b) inactive socialising (sitting and talking, talking on the phone, playing board games, etc.);

(c) loco-play (time spent in active locomotion and unorganised play, such as playground games, etc.); and

(d) sport (physical activities with recognised rules, governing associations and typically taking place in specialised spaces, such as ovals, courts, etc.).

It was expected that the following age trends would be displayed: activity level (as expressed as PAL) would decrease with age [36,37]; declines would be most pronounced in MVPA and play [26,36-38]; inactive socialising would increase as play decreased [38]; and screen-based activities would increase with age [36,38]. In addition, the following gender relationships were expected: boys would be more active than girls [37,38]; boys would spend more time playing sport [37,38]; girls would spend more time in inactive social activities [26] and; boys would spend more time in screen-based activities [26].

## Results

### Sample characteristics

The sample sizes, gender, mean (SD) age, and BMI of the children involved in the test-retest reliability, criterion validity and content/construct validity studies are shown in Table 1. There were no differences between male and female participants in age or BMI.

### Test-retest reliability

**Figure 3:**
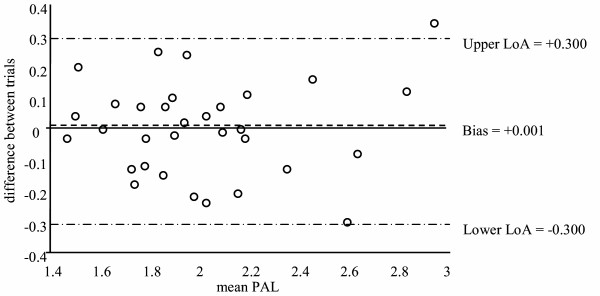
**Bland-Altman plot (bias and limits of agreement) for the test-retest values for physical activity level (PAL) when the MARCA was administered twice (285 min apart) during one day**. Note: LoA = limits of agreement, PAL = physical activity level (a weighted mean MET.d^-1 ^score)

Table 2 displays the results of the reliability analyses. Test-retest ICCs showed high reliability for all three MARCA variables. Paired t-tests revealed no significant differences between reported activity over the two administrations. However, Bland-Altman analyses revealed wide limits of agreement for each of the MARCA variables. The Bland-Altman plot for PAL is shown in Figure 3.

**Table 2:** Intra-class coefficients (ICC), mean values across 2 administrations, and Bland-Altman analyses assessing the reliability of three MARCA variables.

**Variable**	**ICC**	**Mean**	**Bias**	**SEM**	**Upper LoA**	**Lower LoA**
PAL	0.93	2.02	+0.001	0.11	+0.30	-0.30
MVPA min	0.94	196.2	-1.1	18.9	+51.2	-53.4
locomotion min	0.88	193.1	+6.9	26.1	+79.2	-65.4

Note: n = 32; ICC = intra-class coefficient; SEM = standard error of measurement (SD/√2); LoA = limit of agreement; PAL = physical activity level (a weighted mean MET.d^-1 ^score); MVPA = minutes spent in moderate to vigorous physical activity; locomotion = minutes spent in locomotion.

### Criterion validity

Table 3 lists descriptive data for the MARCA and Actigraph variables. The MARCA variables listed in Table 3 were calculated for the entire day, not the Actigraph monitored times (mean = 600 min) that were used in the validity coefficient analyses to allow for comparison across studies. Unpaired t-tests revealed a significant difference in counts.min^-1 ^between genders (boys > girls, p = 0.01). No differences were found between genders for total Actigraph counts or any of the MARCA variables. Table 4 displays the validity coefficients (Spearman rho) for each of the MARCA variables and the Actigraph counts. The validity coefficients were greater in females than males and greater in children aged > 11 years.

**Table 3:** Descriptive data for the MARCA and the Actigraph variables.

**Variable**	**All **	**Male **	**Female **
PAL	1.87	1.88	1.87
	*0.29*	*0.27*	*0.31*
MVPA min.d^-1^	168.7	178.0	159.3
	*73.3*	*69.6*	*76.7*
locomotion min.d^-1^	159.5	167.4	151.7
	*76.8*	*71.9*	*81.7*
total Actigraph counts	458327	475048	441606
(mean = 600 min)	*131627*	*104695*	*153801*
Actigraph counts.min^-1^	766	816*	716*
	*207*	*164*	*234*

Note: n = 66; Upper value = mean; lower value = standard deviation; *significant difference between genders: p < 0.05; PAL = physical activity level (a weighted mean MET.d^-1 ^score); MVPA = minutes spent in moderate to vigorous physical activity across the entire day; locomotion = minutes spent in locomotion across the entire day.

**Table 4:** Spearman correlations between MARCA variables and Actigraph counts for all children and sub-sets of gender and age (<11 y and >11 y).

**Variable**	**All**	**Male**	**Female**	**< 11 y**	**≥ 11 y**
	**rho**	**rho**	**rho**	**rho**	**rho**
	***n***	***n***	***n***	***n***	***n***
PAL	0.45**	0.34	0.62**	-0.1	0.57***
	*66*	*33*	*33*	*15*	*51*
MVPA	0.35**	0.18	0.43*	0.21	0.41**
	*66*	*33*	*33*	*15*	*51*
locomotion	0.37**	0.14	0.59***	0.18	0.43**
	*66*	*33*	*33*	*15*	*51*

Note: Upper value = Spearman rho; lower value = sample size (n); *p < 0.05; **p < 0.01; ***p < 0.001; PAL = physical activity level (a weighted mean MET.d^-1 ^score); MVPA = minutes spent in moderate to vigorous physical activity; locomotion = minutes spent in locomotion.

### Content/construct validity

**Figure 4:**
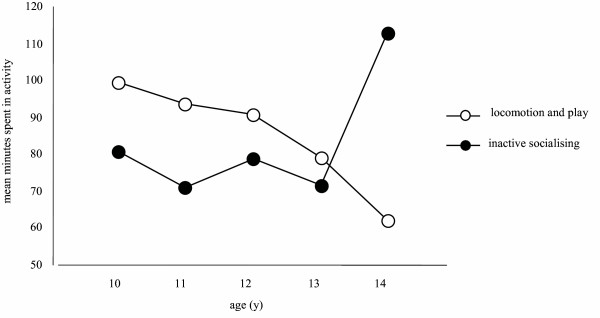
**Change in minutes spent in loco-play compared to the change in minutes spent in inactive socialising with increasing age in years**. Note: There were similar proportions of males and females in each age group.

Table 5 shows the percentage increase or decrease (based on the regression coefficient) per year for PAL, MVPA and total minutes spent in each activity category for all participants in the large content/construct validity sample. Activity variables that displayed significant linear relationships with age are noted with asterisks. Table 6 compares PAL, MVPA and mean time spent in each of the activity variables in males and females. Figure 4 shows the decrease in minutes spent in loco-play with age compared to the increase in minutes spent in inactive socialising with age.

**Table 5:** Approximate percent change in PAL, MVPA and activity variables for every year increase in age.

**Variable**	**Mean rates of change (approximate % change per year)**
PAL	-2.5%***
MVPA	-4.8%**
screen	+10.2%**
inactive socialising	+8.1%*
loco-play	-8.2%**
sport	-7.7%**

Note: n = 1429; those variables exhibiting significant linear relationships with age are noted with asterisks, *p < 0.05; **p < 0.01; ***p < 0.001; PAL = physical activity level (a weighted mean MET.d^-1 ^score); MVPA = moderate to vigorous physical activity; age range = 9.1–15.3 y; screen time = time spent watching television, playing video games and using computers.

**Table 6:** Mean values and percentage differences for PAL, MVPA and time spent in each activity variable.

**Variable**	**Male**	**Female**	**% difference (male - female)/female**
PAL	1.73	1.61***	+7
MVPA min	152.5	121.7***	+25
screen min	273.7	203.8***	+35
inactive socialising min	61.9	91.2***	-33
loco/play min	87.6	91.5	-4
sport min	70.0	38.4***	+75

Note: Significant differences between genders are noted with asterisks; *p < 0.05; **p < 0.01; ***p < 0.001; PAL = physical activity level (a weighted mean MET.d^-1 ^score); MVPA = moderate to vigorous physical activity; min = minutes spent across the entire day.

## Discussion

The MARCA showed high reliability as assessed by ICCs and paired t-tests. This is consistent with a number of studies that report reliability coefficients of r > 0.90 in 1-d self-report questionnaires [5]. Despite the high reliability coefficients, the Bland-Altman analyses revealed relatively wide variation in limits of agreement, suggesting the MARCA is more reliable for group physical activity estimates than individual estimates. However as few studies have reported reliability using Bland-Altman analyses, comparisons across questionnaires is difficult.

The MARCA has comparable validity to other 1-d self-report questionnaires, in particular the MARCA reports similar validity coefficients to other multimedia self-report questionnaires [5,20,39]. High validity coefficients in multimedia self-report questionnaires may be due to tools, such as graphics and sound, assisting children to recall their physical activity. In addition, anomalous data are reduced due to the availability of automatic checks and warnings. The validity analyses conducted on gender and age subsets revealed that validity was higher in girls and children aged ≥ 11 years. While these data should be interpreted with caution due to low sample numbers, especially for children < 11 y, they provide support for investigating the likely relationships between age, gender and validity coefficients of self-report questionnaires.

The MARCA exhibits content validity as it provides information on various sedentary behaviours, as well as moderate and vigorous physical activities, that each contribute to health outcomes. Further, the MARCA is capable of investigating other subsets of activity, such as screen-based activities and inactive socialising, which may be related to health outcomes and display trends with increasing age [7,38].

As expected, the construct validity analyses revealed activity level decreased significantly with age. The rate of change was larger in MVPA than overall PAL. The rate of change was slightly larger in play than in sporting activities. As predicted, the pattern of decrease in time spent in play with increasing age was mirrored by an increase in inactive socialising [38]. This is likely to be reflective of the complex psychological, physical and social developmental changes that occur post-puberty [40], such as the increased importance of peer support and social networks. Boys were also shown to be more active than girls. Boys spent more time in sport and screen-based activities, suggesting they may be more polarised in their activity behaviours, moving from MVPA to sedentary behaviour with little time spent in low-level activity. In contrast, girls spent significantly more time in low-level activities; inactive socialising. As these results are consistent with a number of investigations into change in activity level with increasing age [26,36-38] and difference in activity between genders [26,37,38], the MARCA appears to show good construct validity.

## Conclusion

The MARCA has been found to be a reliable self-report instrument that exhibits good content and construct validity. In addition, the criterion validity of the MARCA was found to be comparable to the most valid and commonly used existing 1-d self-report questionnaires [14,15,19,39]. As is the case for all self-report validation studies, the MARCA was limited by the lack of a 'gold standard' criterion measure. The reliability and validity studies were also limited by small sample sizes, which restrict the amount of generalisation that can be made about the MARCA's reliability and validity across a wide range of ages and diverse groups of children. To ensure greater confidence about the validity of the MARCA, further criterion validity studies should be undertaken using a wider range of comparative 'criterion' measures.

The poor validity exhibited in existing self-report questionnaires, along with a lack of sensitivity to detect trends and relationships, may be partially responsible for the numerous equivocal findings concerning relationships between: physical activity and health; trends in physical activity and sedentary behaviour; and the correlates of these behaviours. The MARCA offers significant advantages over existing self-report questionnaires, due to the richness of the self-report data collected and its diverse analytical capabilities. Furthermore, the MARCA is capable of collecting self-report data on a wide range of both physical activities and sedentary behaviours. A unique feature of the MARCA is the integration of a compendium of energy costs that contains child-specific data where available. In addition, the MARCA's analytical capabilities considerably reduce researcher burden related to data entry, data cleaning and data analysis. The MARCA can be administered to large numbers of subjects using school computer pools, etc. Computer accessibility may be a possible limitation to administration in some studies, particularly those where a research design calls for multiple administrations. However, due to the expansion of the technological world it is likely that the number of computers in schools and homes will increase, therefore improving the feasibility of large scale studies using computer-delivered instruments. Future research will also focus on the efficacy of recalling two or more day's activity during a single administration of the MARCA, in an attempt to reduce both subject and research burden.

As the trends of increasing overweight and obesity appear to be escalating, future research will attempt to resolve some of these issues. In order to do this, research will need to address all types of activity, not just MVPA. In particular, investigation into sedentary behaviour is likely to be an emerging research area. Use-of-time instruments that provide information on the diverse range of activities during various times of day may be the key to resolving some of the unanswered questions. Therefore during these future investigations, measures such as the MARCA will play an integral role in ensuring the physical activity and sedentary behaviour data collected are accurate enough to reveal important trends and relationships.

## Competing interests

The author(s) declare that they have no competing interests.

## Authors' contributions

KR directed all aspects of the study, including co-development of the MARCA, design of the study, administration of the reliability and validity studies and analyses. KR led the writing of the manuscript. TSO co-developed the MARCA and helped with study design, analyses and assisted with writing of the manuscript. AH contributed to the design and administration of the validity and reliability studies and analyses, and assisted in writing of the manuscript.

## Supplementary Material

Additional File 1 **MARCA moderate intensity video**. The following .mov file is an example of the videos played when subjects select that help is required to determine a subjective intensity ranking for their chosen activity. The .mov file can be played through any application that supports .mov files (e.g. Quicktime and Microsoft Windows Player). Click here for file
